# Wnt/β-Catenin Signaling Regulates the Expression of the Ammonium Permease Gene *RHBG* in Human Cancer Cells

**DOI:** 10.1371/journal.pone.0128683

**Published:** 2015-06-01

**Authors:** Ahmad Merhi, Christelle De Mees, Rami Abdo, Jennifer Victoria Alberola, Anna Maria Marini

**Affiliations:** Biology of Membrane Transport Laboratory, IBMM, Université Libre de Bruxelles, Gosselies, Belgium; University of Kentucky, UNITED STATES

## Abstract

Ammonium is a metabolic waste product mainly detoxified by the liver. Hepatic dysfunction can lead to cytotoxic accumulation of circulating ammonium and to subsequent encephalopathy. Transmembrane ammonium transport is a widely spread process ensured by the highly conserved proteins of the Mep-Amt-Rh superfamily, including the mammalian Rhesus (Rh) factors. The regulatory mechanisms involved in the control of *RH* genes expression remain poorly studied. Here we addressed the expression regulation of one of these factors, *RHBG*. We identify HepG2 hepatocellular carcinoma cells and SW480 colon adenocarcinoma cells as expressing *RHBG* and show that its expression relies on β-catenin signaling. siRNA-mediated β-catenin knockdown resulted in significant reduction of *RHBG* mRNA in both cell lines. Pharmaceutical inhibition of the TCF4/β-catenin interaction or knockdown of the transcription factor TCF4 also downregulated *RHBG* expression. We identify a minimal *RHBG* regulatory sequence displaying a promoter activity and show that β-catenin and TCF4 bind to this fragment *in vivo*. We finally characterize the role of potential TCF4 binding sites in *RHBG* regulation. Taken together, our results indicate *RHBG* expression as a direct target of β-catenin regulation, a pathway frequently deregulated in many cancers and associated with tumorigenesis.

## Introduction

Ammonium, hereafter referring to the sum of the NH_3_ and NH_4_^+^ molecular species, serves as principal nitrogen source for micro-organisms and plants [1]. It is however mostly described for the cytotoxic consequences of its accumulation in animals [2]. Hepatic metabolism of ammonium towards urea and glutamine synthesis is critical to maintain a low plasmatic level of the ammonium emerging from the catabolism of proteins and the activity of the intestinal flora. The impairment of ammonium detoxification occurring in case of liver dysfunction can lead to the development of hepatic encephalopathy and, in acute cases, to lethal cerebral paralysis. In parallel to these toxic effects, renal ammonium production from glutaminolysis and its subsequent urinary excretion is a crucial process to ensure blood pH homeostasis [3]. The view that the transmembrane fluxes of ammonium are the sole consequence of NH_3_ free diffusion was held for decades, till the first genes encoding specific ammonium permeases were identified [4–6]. Sequence analysis enabled to define a new and widely conserved family of proteins termed Mep-Amt-Rh, represented in vertebrates by the well known human Rhesus blood group factors [7]. Non-erythroid Rh factors, RhBG and RhCG, were subsequently discovered and notably found to be expressed in specific epithelial cells of several organs including mouse and human liver and kidney [8–14]. Mice knockout studies revealed the role of Rhbg and Rhcg in renal urinary ammonium excretion while their potential involvement in the liver physiology remains unsolved [15–17]. Of note, *RHBG* appears overexpressed in human hepatocellular carcinoma bearing activating mutations in β-catenin [18], suggesting a correlation between Wnt/β-catenin signaling and human *RHBG* regulation. This correlation appears to hold true in a normal mouse liver context as transgenic models enabling targeted inactivation or activation of β-catenin signaling show downregulation or upregulation of *RHbg* expression, respectively [19,20]. The Wnt/β-catenin pathway is highly conserved across metazoans and regulates cell proliferation, differentiation, and survival [21–23]. The secreted proteins of the WNT family are able to bind specific Frizzeld/Lrp receptors and activate signal transduction via different mechanisms. In the canonical Wnt/β-catenin mechanism, absence of Wnt signaling is accompanied by a low cytosolic β-catenin level. The β-catenin stability is regulated by a destruction complex, formed by axin, adenomatous polyposis complex (APC), casein kinase 1 and glycogen synthase kinase-3β (GSK-3 β) that phosphorylates β-catenin at its N-terminus and leads to its ubiquitylation and subsequent proteasomal degradation [21,22]. Wnt signaling triggers the dissociation of the β-catenin/destruction complex [23–25]. The resulting inhibition of phosphorylation leads to cytosolic β-catenin accumulation and translocation into the nucleus. Beta-catenin can then activate the transcription of various target genes as a cofactor bound to members of the T-cell factor (TCF)/lymphoid enhancer factor (LEF) transcription factor family and drive Wnt-specific transcriptional programs [23]. Abnormal activation of Wnt/β-catenin signaling, due to loss-of-function mutations in APC or activating mutations in β-catenin has been linked to tumorigenesis in many settings including melanoma, breast, colon and hepatocellular carcinomas [21,23].

The regulatory mechanisms involved in the control of human *RH* genes expression and the signaling pathways potentially implicated are so far poorly documented. Here, we sought to identify human cancer cell lines expressing the *RHBG* gene to study its expression regulation and address the potential direct influence of the Wnt/β-catenin signaling. We show that *RHBG* is highly expressed in HepG2 hepatoma cells and relies on β-catenin signaling. Similarly, the *RHBG* expression revealed in SW480 colon cancer cells is dependent on β-catenin, further supporting the role of β-catenin signaling in *RHBG* regulation. Promoter analysis and chromatin immunoprecipitation assays are consistent with a direct involvement of TCF4/β-catenin in *RHBG* up-regulation in HepG2 cells.

## Materials and Methods

### Cell culture and reagents

The human hepatocellular carcinoma (HepG2 and Hep3B) and human embryonic kidney (HEK293T) cell lines were kindly provided by Professor Claude Szpirer, Université Libre de Bruxelles, Belgium. The human colon adenocarcinoma (SW480) cell line was purchased from CLS (Germany). HepG2, Hep3B, SW480 and HEK293T cells were cultured in advanced DMEM medium (Invitrogen) supplemented with 10% fetal bovine serum (Biowest), 2 mM L-glutamine, 50 units/ml penicillin, and 50 μg/ml streptomycin. Cells were maintained in an incubator with humidified air (5% CO2) at 37°C. PFK118-310 (#K4394) was purchased from Sigma and used at a 0,2 or 0,4μM concentration from a DMSO solution.

### Plasmids construction

DNA fragment (2492 bp) corresponding to the potential *RHBG* promoter was amplified using polymerase chain reaction with the F-Pr- BG- and R-Pr-BG (Table 1) primers and the human genomic DNA of HEK293T cells as a template. The PCR product was digested with SacI and BglII restriction enzymes and cloned into pGL3-Basic (Promega) that has been linearized with the same restriction enzymes. Deletion mutants were then constructed using the pGL3-RHBG plasmid as a template and the indicated primers (Table 1). The PCR products were digested with SacI and BglII restriction enzymes and cloned into pGL3-Basic. All constructs were verified by sequencing.

**Table 1:** Primer sequences used for cloning.

RHBG	Forward	CCCGAGCTCCAGTCCCTGATTGGACAGCC
	Reverse	CCCAGATCTGCGGACAAAGACAGCAAAGA
Mut-A	Forward	CCCGAGCTCACCTGAGTTCCTGAACACAC
Mut-B	Forward	CCCGAGCTCACCAGACAGTGGTTCCTGGA
Mut-C	Forward	CCCGAGCTCTGGAGAGCACTATTTATCTC
Mut-D	Forward	CCCGAGCTCAACATCATGTATGAGGGTG
Mut-E	Forward	CCCGAGCTCTATCAGGGCAGAACACATGG
Mut-F	Forward	CCCGAGCTCTTTCCAGGGTCGCGTCCAA
Mut-G	Forward	CCCGAGCTCCGCCCCGCTCCTG
Mut-H	Forward	CCCGAGCTCAGCAGGGCCTCTGTGG
Mut-I	Forward	CCCGAGCTCTGCCGCCGGGAATTGTC
Mut-J	Forward	CCCGAGCTCCTGCGAGCGCCAGCCGAGA
ΔTCF3	Reverse	CCCAGATCTACAGCAAAGAGGACGGCAGTG
ΔTCF2, 3	Reverse	CCCAGATCTCGGCAGTGGCGCCCTGGAG

### RNA extraction and qRT-PCR

Total cellular RNA was extracted using TRIzol reagent (Invitrogen) according to manufacturer instructions. DNase treatment was done using a DNA Removal Kit (Invitrogen, #AM1906). One μg of total RNA was reverse-transcribed to cDNA using the SuperScriptIII First-Strand Synthesis SuperMix (Invitrogen) according to manufacturer instructions. Realtime RT-PCR were performed on a StepOnePlus Real-Time PCR System (Applied Biosystems) using GoTaq qPCR Master Mix (Promega) using the indicated primers (Table 2) and normalized to β-actin mRNA level measured in parallel.

**Table 2:** Primer sequences used for qRT-PCR.

RHBG	Forward	CCTCAAGTGAAATGATGCTG
	Reverse	ATTTTGATTCAAGGATGGGC
RHCG	Forward	AGTCTATGGAAAAGAAGGGC
	Reverse	CACCAAGAGACCATAAATCTG
ACTIN B	Forward	CTGGAACGGTGAAGGTGACA
	Reverse	AAGGGACTTCCTGTAACAATGCA
β- CATENIN	Forward	CAACTAAACAGGAAGGGATG
	Reverse	CACAGGTGACCACATTTATATC
TCF4	Forward	AAGAGCAAGCGAAATACTAC
	Reverse	CTTCTTTCCATAGTTATCCCG
GLUL	Forward	GTGAAGACTTTGGAGTGATAG
	Reverse	GATGTACTTCAGACCATTCTC
CYLCIN D1	Forward	GCCTCTAAGATGAAGGAGAC
	Reverse	CCATTTGCAGCAGCTC
AXIN2	Forward	AAAGAGAGGAGGTTCAGATG
	Reverse	CTGAGTCTGGGAATTTTTCTTC
RHBG-CHIP	Forward	ATTGTCTGCCAAAGCCTGCG
	Reverse	AGACAGCAAAGAGGACGGC
AXIN2-CHIP	Forward	TGCTTGCCACTGTTTGAAGTCAG
	Reverse	GCCATGAACCCTTTTTGATCTTGC

### Immunofluorescence staining

Cells were cultured in Millicell EZ 8 chamber slide (Millipore). Cells were fixed with 4% paraformaldehyde (PFA) for 15 min and subsequently permeabilized with 0,1% TritonX-100 for 3 minutes and were next blocked with goat serum (5%) for 30 minutes. Cells were incubated with β-catenin antibody (Cell signaling #8480, 1:100 in 5% goat serum) overnight at 4°C. Slides were washed and incubated with anti-rabbit IgG (H+L), F(ab')2 Fragment (Alexa Fluor 594 Conjugate, Cell signaling, 1:250 in 5% goat serum) for 1 hour at room temperature. After washing with PBS, the slides were mounted with ProLong Gold antifade reagent with DAPI (Invitrogen).

### Western Blot analysis

Total proteins were extracted using RIPA (25mM Tris-HCl pH 7.6, 150mM NaCl, 1% NP-40, 1% sodium deoxycholate, 0.1% SDS) lysis buffer supplemented with cocktail of phosphatase and protease inhibitors (Roche). After centrifugation, proteins were quantified using Pierce Microplate BCA Protein Assay Kit—Reducing Agent Compatible assay (Thermo Scientific). Equal amounts (~20μg) of proteins were then separated by Mini-PROTEAN TGX gels (Bio-Rad). Proteins were transferred to nitrocellulose membrane (Protran, VWR). Membranes were blocked with 5% milk and incubated with anti-β-catenin (Cell signaling, #8480, 1:1000), anti-TCF4 (#2569, 1:1000), anti-Glutamine synthetase (Abcam, #ab73593, 1:1000) or anti-β-Actin (Sigma, #A2228, 1:2000). Primary antibodies were detected with horseradish-peroxidase-conjugated anti-rabbit or anti-mouse-IgG secondary antibodies (GE Healthcare) followed by measurement of chemoluminescence (Lumi-LightPLUS, Roche).

### RNA interference

Cells were reverse transfected with pre-designed silencer select non-targeting control (Invitrogen, #4390843) or siRNAs targeting β-catenin (Invitrogen, #s438) or TCF4 (Invitrogen, #s13880) with Lipofectamine siRNAMAX (Invitrogen). Cells were incubated at 37°C for 72 or 96 hours and the indicated mRNA and proteins level were examined by qRT-PCR and western blotting, respectively.

### Transfection and luciferase assay

Cells were transiently co-transfected with the pTK-Renilla luciferase reporter vector (Promega) and empty plasmid (pGL3-Basic, Promega) or the plasmid containing the indicated *RHBG* promoter constructs for 48h using the Viafect transfection reagent (Promega). Luciferase activity in total cell lysates was measured using the Dual-Glo luciferase reporter assay (Promega) according to manufacturer instructions. Luminescence was measured using GloMax-96 Microplate Luminometer (Promega).

### Chromatin immunoprecipitation (CHIP) assay

CHIP assay was done using SimpleChIP Enzymatic Chromatin IP Kit (Magnetic Beads, Cell signaling) according to manufacturer instructions. Briefly, cells were fixed with 1% formaldehyde solution to cross-link histone and non-histone proteins to DNA. Nuclear chromatin was digested with Micrococcal Nuclease for 20 min at 37°C and then incubated overnight at 4°C with either anti-β-catenin (Cell signaling, #8480, 1:50), anti-TCF4 (#2569, 1:50) Normal Rabbit IgG (Cell signaling, #2729). Following washing with low and high salt ChIP buffers, the protein-DNA complexes were eluted and cross-links were then reversed. After proteinase K digestion, DNA is purified and quantified by Real time-PCR as described earlier using primers listed in Table 2 and designed to amplify the indicated promoter regions of the target genes.

### Statistical analysis

Data are expressed as means ± S.E.M. Statistical comparisons were assessed by Student’s *t*-tests using Graph Pad Prism version 5.00 software (Graph Pad Software). Differences were considered significant when the p value is below 0.05 (* p < 0.05, ** p < 0.01, *** p < 0.001), n = 3, except for Chip experiments where n = 2.

## Results

### *RHBG* is highly expressed in HepG2 hepatoma cells

The human *RHBG* gene was found to be overexpressed in a subset of hepatocellular carcinoma [18], however, the regulatory mechanisms involved remain unknown. In order to identify a cancer cell line that could be used to address the regulation of *RHBG*, we evaluated its expression levels in the HepG2 and Hep3B hepatoma cells. HepG2 cells bear a heterozygous deletion in the exon3 of the β-catenin *CTNNB-1* gene, producing a truncated β-catenin protein lacking key residues for its phosphorylation by the destruction complex and thus resulting in its cellular accumulation [26–28]. Hep3B cells are derived from a hepatitis B- infected liver tumor, and do not contain mutations in the β-catenin gene [29,30].

**Fig 1:**
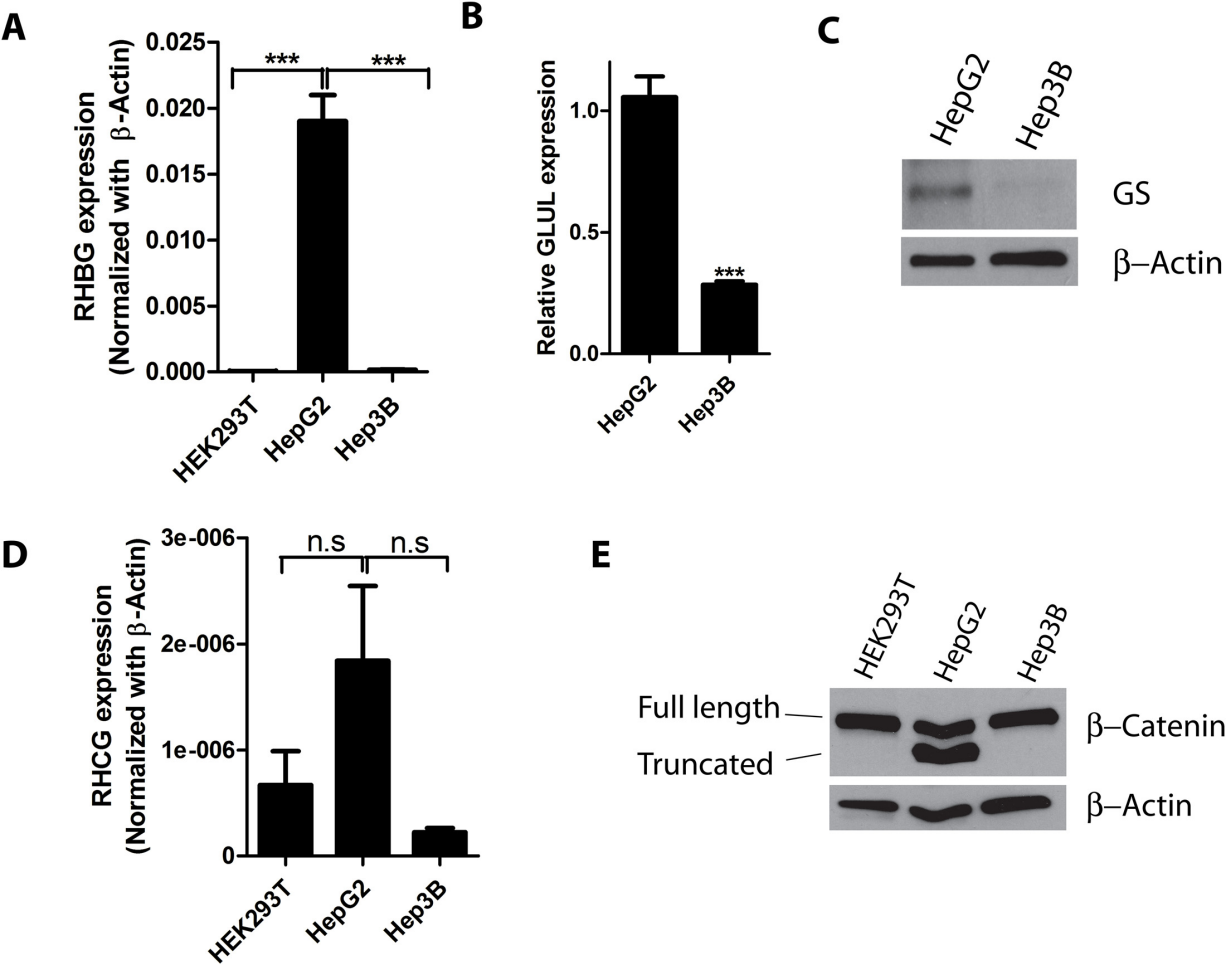
*RHBG* and *GLUL* are highly expressed in HepG2 hepatoma cells. A) The level of mRNA expression of *RHBG* in HEK293T, HepG2 and Hep3B cells was determined by qRT-PCR and normalized to β-actin. B) The mRNA expression of *GLUL* in HepG2 and Hep3B was determined by qRT-PCR. C) Western Blot analysis of GS protein from total cell lysates of HepG2 and Hep3B cells. D) The level of mRNA expression of *RHCG* in HEK293T, HepG2 and Hep3B cells was determined by qRT-PCR and normalized to β-actin. E) Western Blot analysis of β-catenin protein from total cell lysates of HEK293T, HepG2 and Hep3B cells.

The *RHBG* gene appeared highly expressed in HepG2 compared to Hep3B cells, the latter showing slightly higher expression level compared to that of HEK293T cells taken as a normal cell model (Fig 1A). Expression level of *GLUL*, the gene of glutamine synthetase (GS) was also upregulated in HepG2 cells compared to Hep3B (Fig 1B). As the GS gene is a reported target of β-catenin [31,32], this could be consistent with a more effective β-catenin signaling in HepG2 cells compared to Hep3B. The *GLUL* expression levels were corroborated by the resulting GS protein levels which appeared higher in HepG2 compared to Hep3B cells (Fig 1C). Upon checking the expression of the second non-erythroid *RH* gene, *RHCG*, very low levels were found in HepG2, Hep3B and HEK293T cells (Fig 1D). These data thus point to HepG2 cells as suitable for the study of *RHBG* expression.

We next checked the β-catenin protein levels in HepG2, Hep3B and HEK293T cells. Consistent with previous reports [33,34], HepG2 cells harbored two β-catenin species likely corresponding to the wild-type and the truncated forms (Fig 1E). A β-catenin protein with the size of the wild-type form was detected in both HEK293T and Hep3B, with no obvious accumulation in the latter cell line. It was previously shown that β-catenin is present in the nucleus of HepG2 cells in contrast to Hep3B cells [34]. These data suggest a correlation between *RHBG* expression in HepG2 cells and nuclear localization of β-catenin.

### Silencing of β-catenin correlates with *RHBG* down-regulation in HepG2 cells

**Fig 2:**
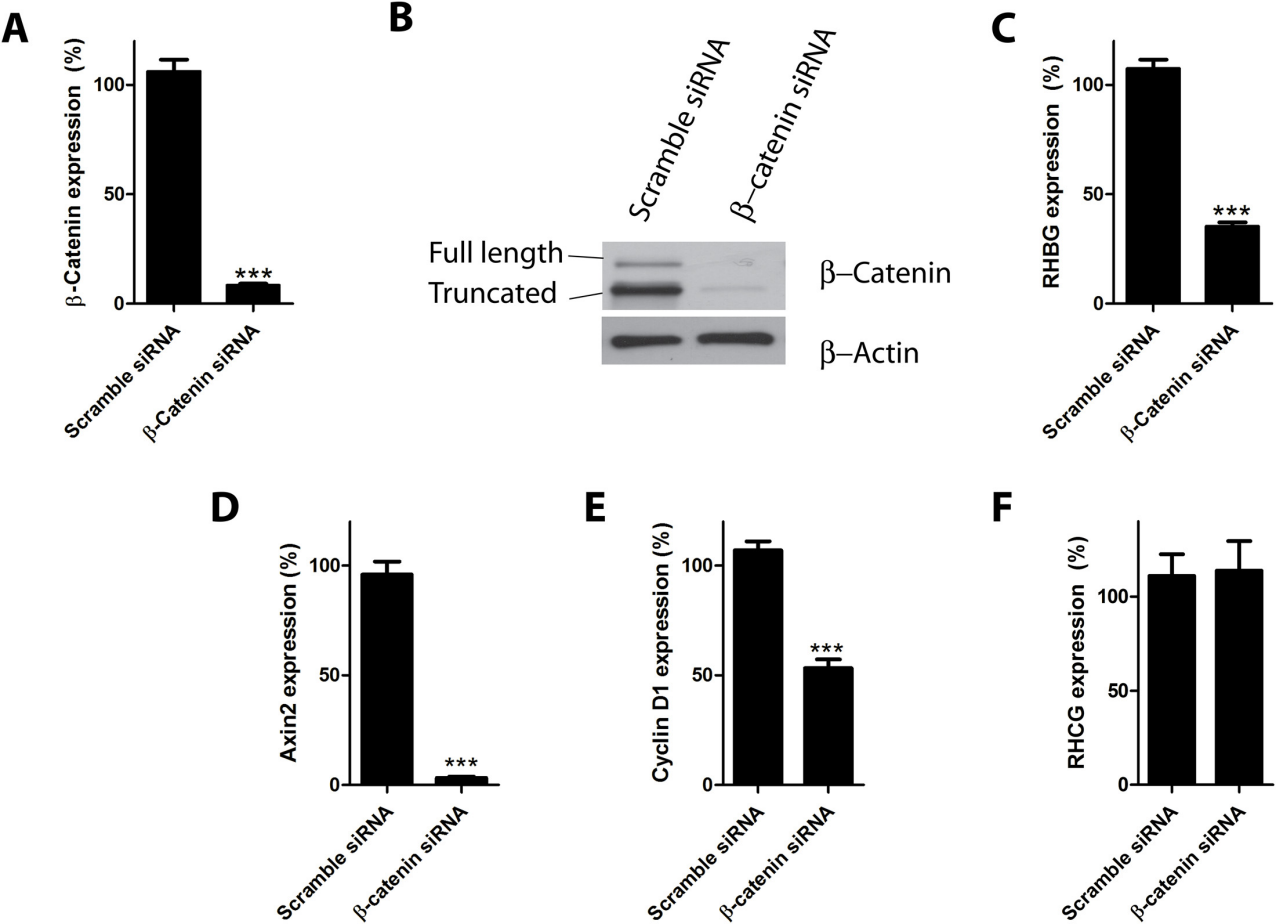
β-catenin knockdown decreases *RHBG* expression in HepG2 hepatoma cells. A-F) HepG2 cells were reverse transfected with β-catenin or control (scramble) siRNAs. 72 hours after transfection, expression levels of *β-catenin* mRNA (A), β-catenin protein (B), *RHBG* mRNA (C), *Axin2* mRNA (D), *Cyclin D1* mRNA (E) and *RHCG* mRNA (F) were determined.

We next used β-catenin siRNA to test whether the *RHBG* gene expression observed in HepG2 cells is related to β-catenin function. Transfection of HepG2 cells with β-catenin siRNA for 72 hours led to a decrease in β-catenin mRNA level compared to cells transfected with non-targeting siRNA (Fig 2A). The reduction of β-catenin mRNA resulted in a reduction of β-catenin protein levels (Fig 2B). Of note, the *RHBG* gene expression was largely reduced upon β-catenin silencing (Fig 2C). Similarly, expression levels of *Axin2* and *Cyclin D1*, two targets of β-catenin regulation [35–38], were also decreased with β-catenin silencing (Fig 2D and 2E). In contrast, the low expression level of *RHCG* observed in HepG2 cells was not affected by β-catenin silencing (Fig 2F). Hence, inhibition of β-catenin is accompanied by the down-regulation of *RHBG* gene expression in HepG2 cells.

### Beta-catenin drives *RHBG* expression in SW480 colon cancer cells

**Fig 3:**
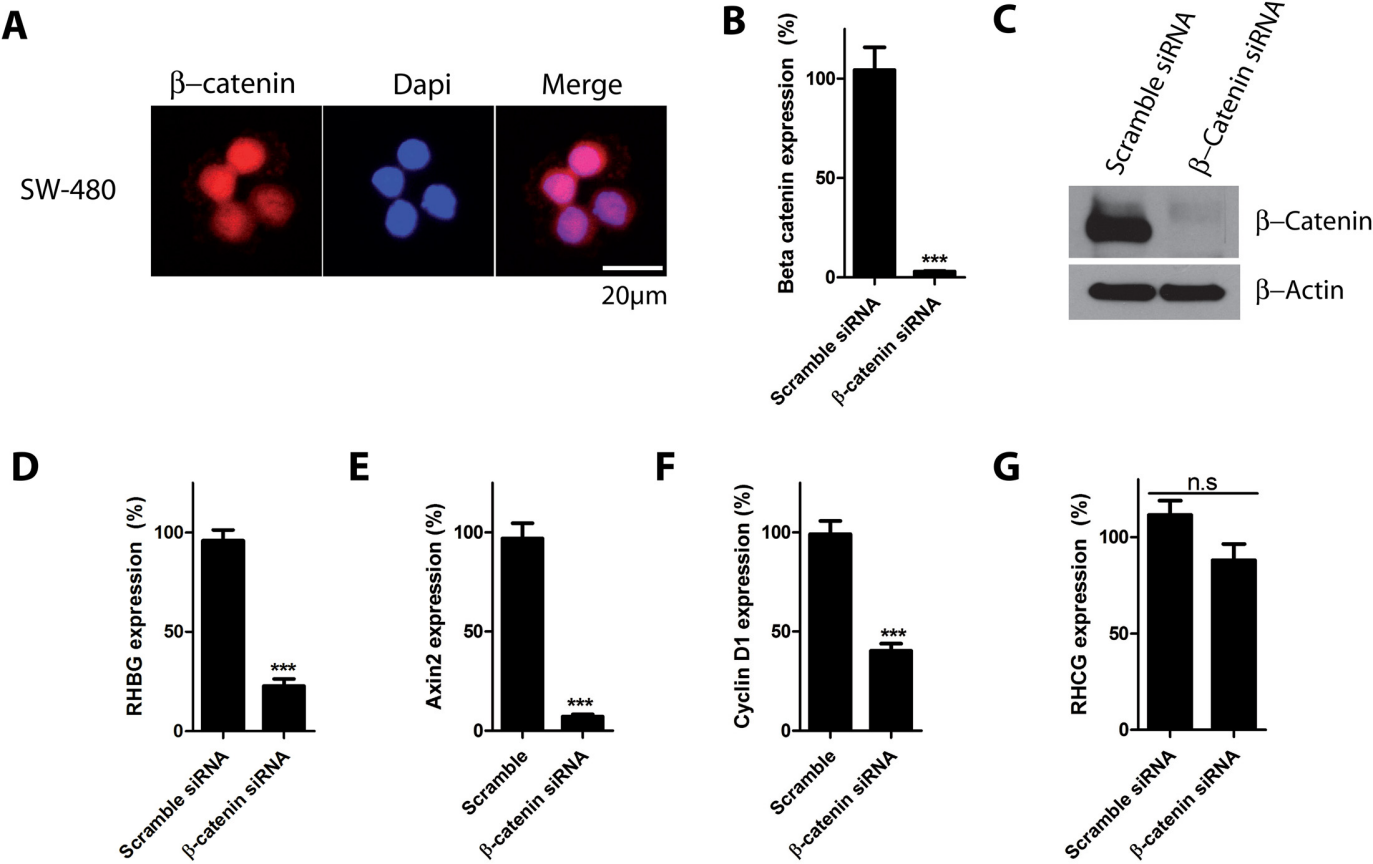
β-catenin knockdown decreases *RHBG* expression in SW480 colon cancer cells. A) Representative results of immunofluorescence of β-catenin localization in SW480 cells using β-catenin antibody. Nuclei were stained with DAPI. B-G) SW480 cells were reverse transfected with β-catenin or control (scramble) siRNAs. 72 hours after transfection, expression levels of *β-catenin* mRNA (B), β-catenin protein (C), *RHBG* mRNA (D), *Axin2* mRNA (E), *Cyclin D1* mRNA (F) and *RHCG* mRNA (G) were determined.

Mutations inducing β-catenin activation have been identified in various types of tumors, including melanoma, prostate, breast, and colon cancers [21,33,39,40]. We next tested whether the β-catenin signaling could be correlated with *RHBG* expression in another cancer cell line harboring β-catenin signaling activating mutations. The SW480 colon cancer cells bear a truncating mutation in the *APC* gene, resulting in stabilization and nuclear accumulation of β-catenin and leading to constitutive activation of β-catenin signaling [41–44]. Consistently, immunofluorescence experiments revealed a strong nuclear localization of β-catenin in these cells (Fig 3A). Transfection of SW480 cells with β-catenin siRNA for 72 hours led to a decrease in both mRNA and protein levels of β-catenin (Fig 3B and 3C). Similarly to HepG2 cells, β-catenin silencing was accompanied by a large decrease in *RHBG* expression in SW480 cells (Fig 3D). The expression levels of *Axin2*, and *CylcinD1* (Fig 3E and 3F) were also decreased upon β-catenin silencing, consistent with β-catenin signaling inhibition. *RHCG* expression was not significantly affected upon β-catenin silencing (Fig 3G).

These results indicate that the correlation between β-catenin signaling and *RHBG* expression can be extended to SW480 colon cancer cells.

### Activation of β-catenin correlates with *RHBG* gene induction in HEK293T cells

**Fig 4:**
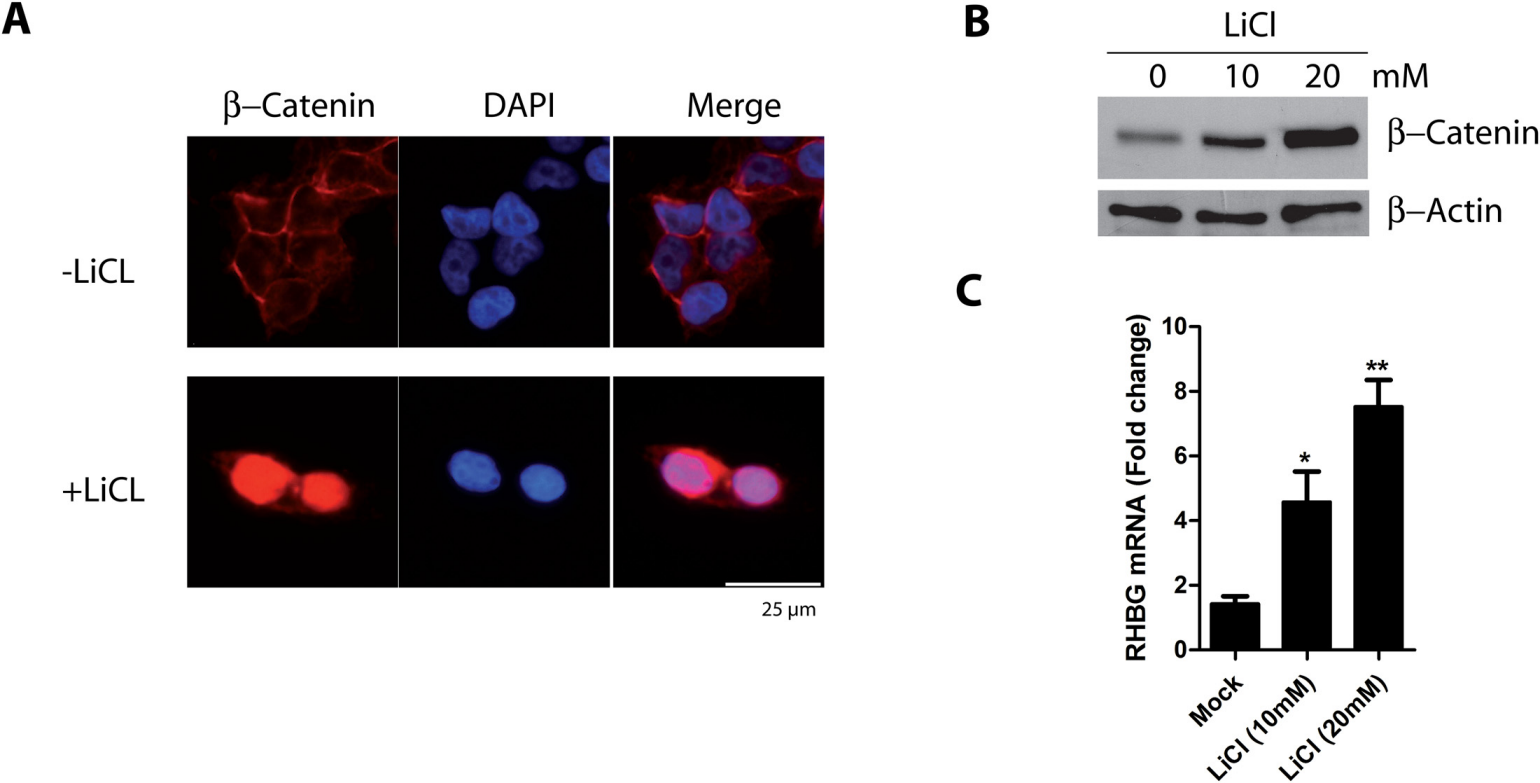
Activation of β-catenin signaling increases *RHBG* expression. A) HEK293T cells were treated with LiCL (20mM) for 24 hours. β-catenin localization was determined by immunofluorescence using β-catenin antibody. Nuclei were stained with DAPI. B) HEK293T cells were treated with LiCL (10 or 20mM) for 24 hours. β-catenin protein level was determined in total cell lysates by immunoblotting using β-catenin antibody. C) Same culture conditions as in B. The *RHBG* mRNA level was determined by qRT-PCR.

We next addressed whether activation of β-catenin in a cell line with no major nuclear activity of β-catenin could be sufficient to induce *RHBG* expression. LiCl is reported to inhibit GSK3 kinase [45,46], leading to β-catenin stabilization and nuclear accumulation [47,48]. We therefore treated HEK293T cells with LiCl (10 and 20 mM) to activate the Wnt/β-catenin pathway. In keeping with previous observations, immunofluorescence and western blot experiments revealed that treatment of HEK293T cells with LiCl for 24 hours induced a nuclear accumulation and stabilization of β-catenin (Fig 4A and 4B). Of note, LiCl treatment concomitantly induced an increase in the mRNA level of *RHBG* in a dose-dependent manner (Fig 4C). This result indicates that *RHBG* expression can be upregulated by artificial activation of β-catenin signaling and suggests that activation of this pathway can be sufficient to induce *RHBG* expression in the HEK293T cells.

### *RHBG* expression in HepG2 cells is dependent on TCF4

**Fig 5:**
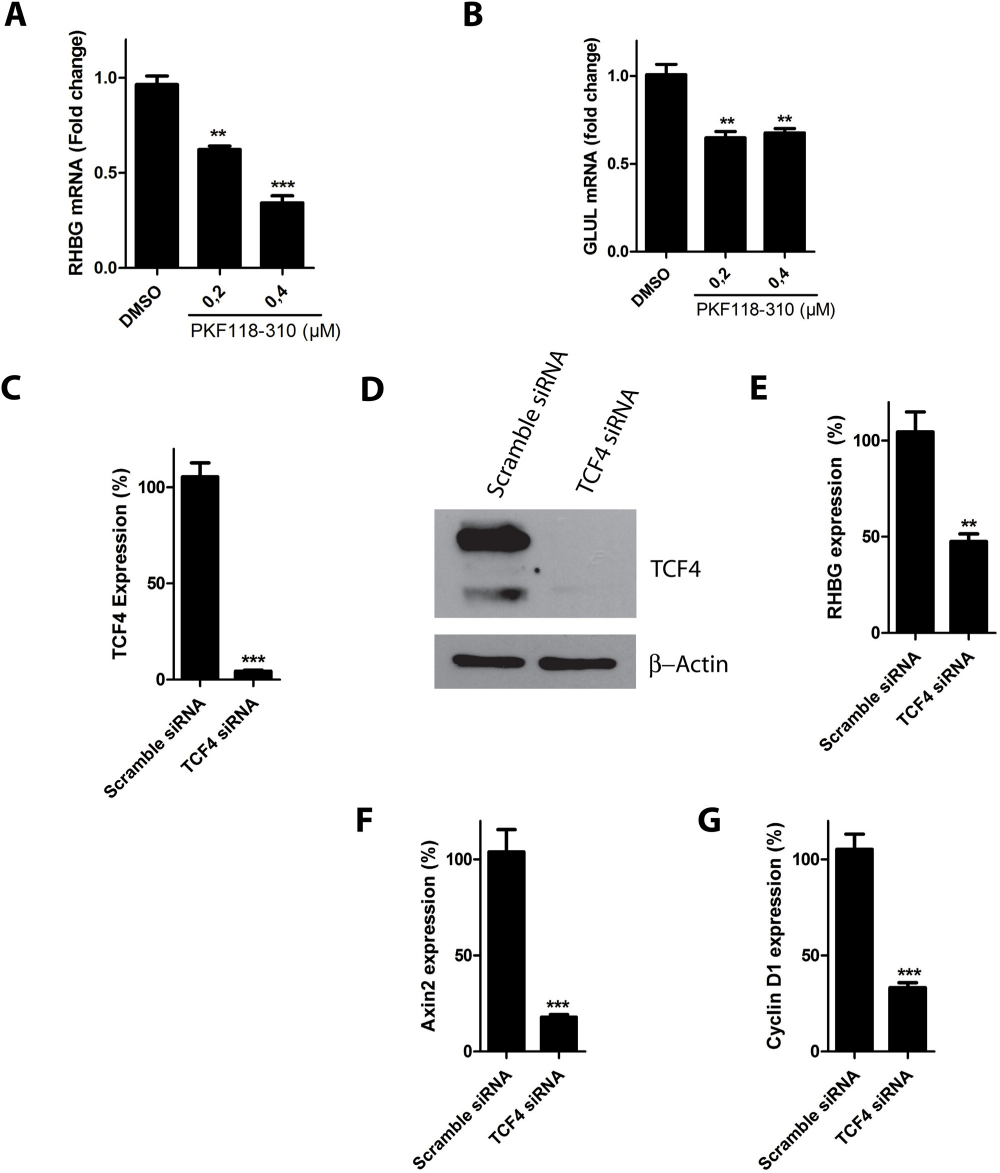
*RHBG* expression is dependent on TCF4. A-B) HepG2 cells were treated with PKF118-310 (0,2 or 0,4 μM) for 24 hours. The *RHBG* (A) and *GLUL* (B) mRNA levels were determined by qRT-PCR. C-G) HepG2 cells were reverse transfected with TCF4 or control (scramble) siRNAs. 72 hours after transfection, levels of *TCF4* mRNA (C), TCF4 protein (D), *RHBG* mRNA (E), *Axin2* mRNA (F), and *Cylcin D1* mRNA (G), were determined.

The Wnt/β-catenin pathway drives Wnt-specific transcriptional programs via the interaction with DNA-binding factors of the TCF/LEF family [21,22]. However, it is reported that β-catenin can also activate gene expression in a TCF4-independent manner [49–51]. To address a role of the TCF4/β-catenin complex in *RHBG* expression, we evaluated the effect of inhibiting the β-catenin activity in HepG2 cells by using an antagonist of the TCF4/β-catenin complex, PKF118-310. This compound disrupts the TCF4/ β-catenin complex and inhibits expression of TCF4-dependent genes [52]. Treatment of HepG2 cells with PKF118-310 was accompanied by a decrease in *RHBG* expression (Fig 5A). The *GLUL* expression level was also decreased by the treatment, consistent with a likely reduction of TCF4/β-catenin mediated transcription in these conditions (Fig 5B).

**Fig 6:**
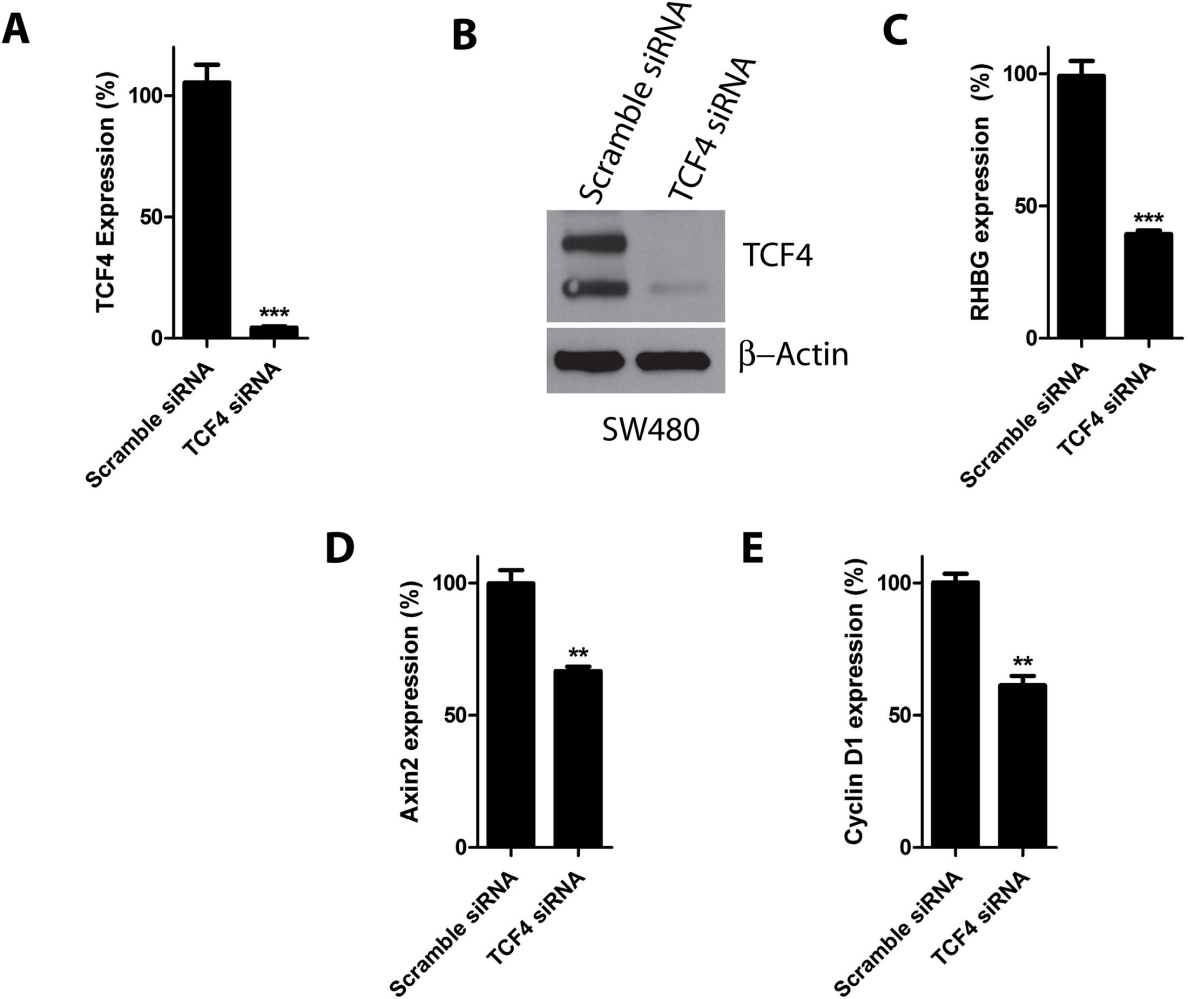
TCF4 knockdown decreases *RHBG* expression in SW480 cells. A-E) SW480 cells were reverse transfected with TCF4 or control (scramble) siRNAs. 72 hours after transfection, levels of *TCF4* mRNA (A), TCF4 protein (B) *RHBG* mRNA (C), *Axin2* mRNA (D), and *Cyclin D1* mRNA (E), were determined.

To further assert a role of TCF4 in *RHBG* expression, we tested the impact of TCF4 knockdown in HepG2 cells. Transfection of the latter cells with TCF4 siRNA for 72 hours decreased both the mRNA and protein levels of TCF4 compared to cells transfected with non-targeting siRNA (Fig 5C and 5D). Importantly, the *RHBG* mRNA level was reduced upon TCF4 silencing (Fig 5E) Similarly, the *Axin2* and *Cyclin D1* mRNA levels were also decreased (Fig 5F and 5G), in keeping with previous observations describing the corresponding genes as targets of TCF4 [38,40]. Moreover, similar TCF4 silencing experiments performed in SW480 colon adenocarcinoma cells also decreased *RHBG*, *Axin2* and *Cyclin D1* mRNA levels (Fig 6A–6E).

These results together indicate that β-catenin-mediated expression of *RHBG* is at least partially TCF4-dependent in both HepG2 and SW480 cells.

### The *RHBG* promoter is activated by β-catenin/TCF4

**Fig 7:**
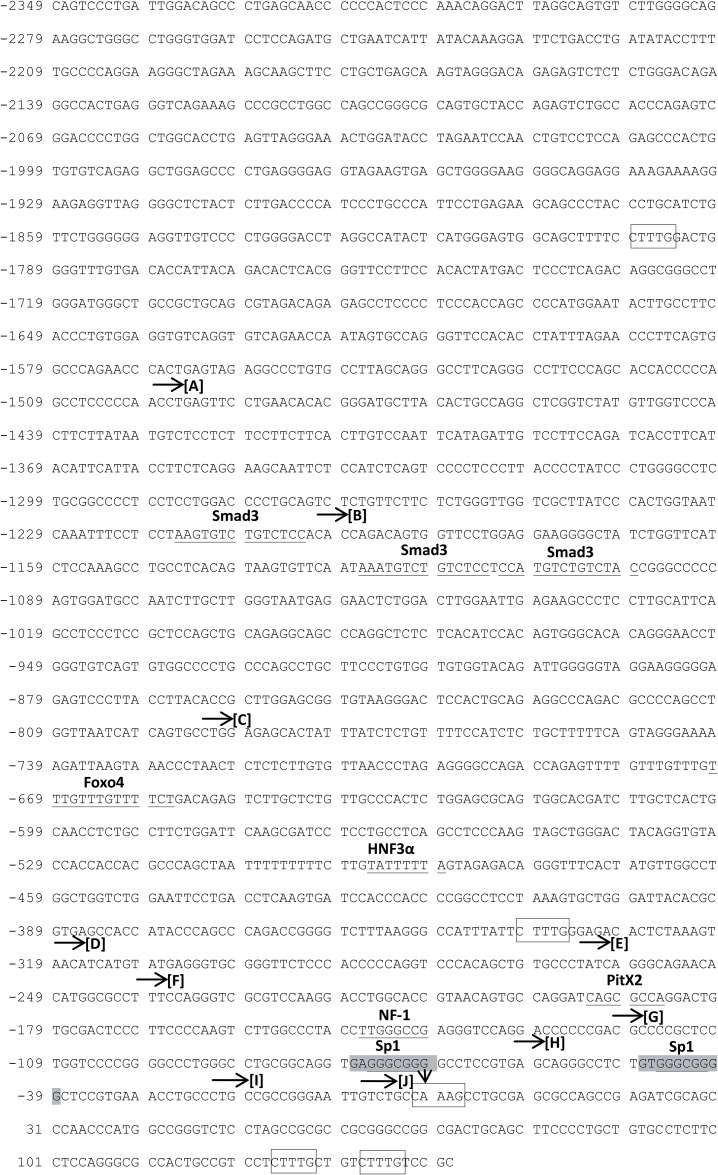
Promoter region of *RHBG* gene. The potential human *RHBG* promoter sequence was obtained from eukaryotic promoter database (http://epd.vital-it.ch/). Black arrow (↓) indicates the predicted transcription start site (TSS) which is designated nucleotide 0. The GC boxes are shadowed. A selection of potential binding sites (with 0 or less than 5% dissimilarity) of transcription factors identified using PROMO [54,55] is underlined. Potential TCF4 binding sites are indicated with empty boxes. Horizontal arrows (→) indicate the starting residue position of each promoter construct analyzed in Fig 8.

**Fig 8:**
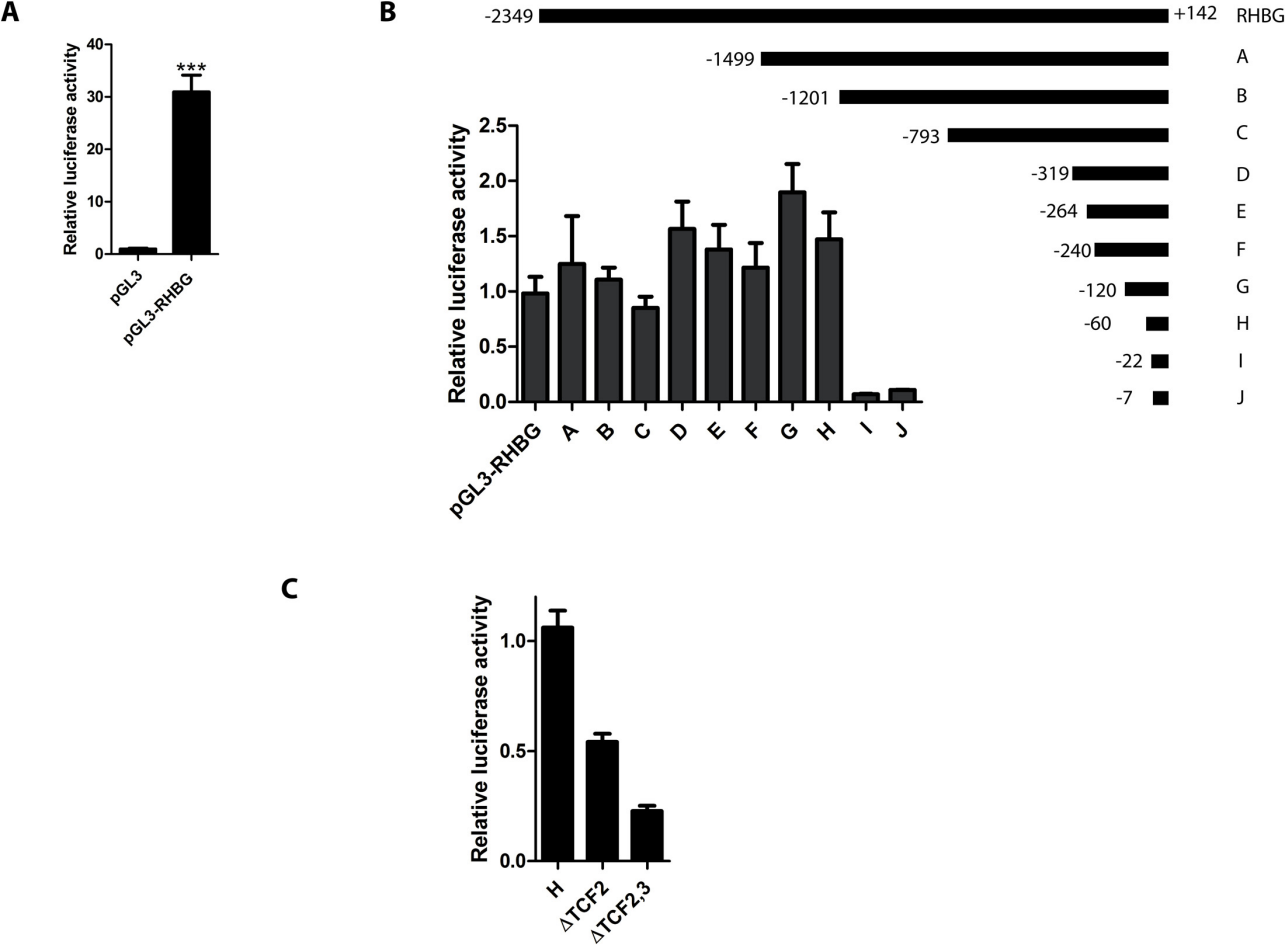
Deletion analysis of *RHBG* promoter sequence. HepG2 cells were transfected with the empty plasmid (pGL3) or *RHBG* promoter (pGL3-RHBG) together with Renilla plasmid. 48 hours after transfection, *RHBG* promoter activity in total cell lysates was determined by luciferase assay. B) HepG2 cells were transfected with *RHBG* promoter (pGL3-RHBG) or the indicated construct together with Renilla plasmid. 48 hours after transfection, *RHBG* promoter activity was determined by measuring luciferase activity in total cell lysates. Data are expressed as mean of triplicate determinations ± S.E.M of the pGL3-RHBG construct relative to pGL3-Basic. C) HepG2 cells were transfected with the indicated construct together with Renilla plasmid. 48 hours after transfection, *RHBG* promoter activity was determined by measuring luciferase activity in total cell lysates.

To further study *RHBG* expression and identify potential regulators, a genomic fragment (Fig 7) containing 2349 bp upstream and 142 bp downstream of the *RHBG* predicted transcriptional start site (TSS) was directionally subcloned into the pGL3-basic firefly luciferase reporter vector. To test whether this fragment possesses a promoter activity, the *RHBG* promoter construct (pGL3-RHBG) and the native pGL3-basic vector were used for transient co-transfection of HepG2 cells together with the pTK-Renilla luciferase reporter vector as transfection control. 48 hours after transfection, the luciferase activity in pGL3-RHBG transfected cells was about 30 fold higher than with the pGL3-basic plasmid indicating that the cloned *RHBG* sequence contains an active promoter (Fig 8A).

Sequence analysis of the *RHBG* regulatory sequence did not reveal a potential TATA box, while the region proximal to the predicted TSS was enriched in G/C content, indicating that the *RHBG* promoter likely corresponds to a TATA-less GC-rich promoter. Two potential GC boxes were depicted, embedded in potential Sp-1 binding sites (Fig 7). To further dissect the regions important for the activity of the cloned *RHBG* promoter, a series of constructs were generated bearing progressive deletions in this DNA fragment (Figs 7 and 8).

Though fluctuations were noted according to the considered fragment, all the constructs containing the -60/+142 region produced a luciferase activity very close or higher than the full-length pGL3-RHBG construct (Fig 8B). Of note the -60/+142 region of fragment H, comprising only one of both GC boxes, retained high luciferase activity. The constructs bearing further 5’ truncation into this region led to a major decrease of the *RHBG* promoter function, the -22/+142 region showing a very low luciferase activity (Fig 8B). This underlines the importance of the DNA segment between fragment H and I, and indicates that the expression impairment is most likely due to the loss of the second GC box. Additionally, analysis of the -60/+142 functional segment revealed the presence of three CTTTG/CAAAG motifs which could serve as TCF4 binding sites (Fig 7). These motifs are either juxtaposed to or downstream of the putative TSS. To evaluate a potential contribution of these motifs to the regulation of *RHBG* gene expression, promoter constructs bearing the -60/+142 region of fragment H with deletion of potential TCF4 binding motif 2, or motifs 2 and 3, were generated. Both constructs showed a promoter activity (Fig 8C). However, deletion of motif 2 reduced the promoter activity to the half of fragment H, and simultaneous deletion of motifs 2 and 3 further decreased the promoter activity, suggesting a contribution of these motifs to the functionality of the *RHBG* promoter.

**Fig 9:**
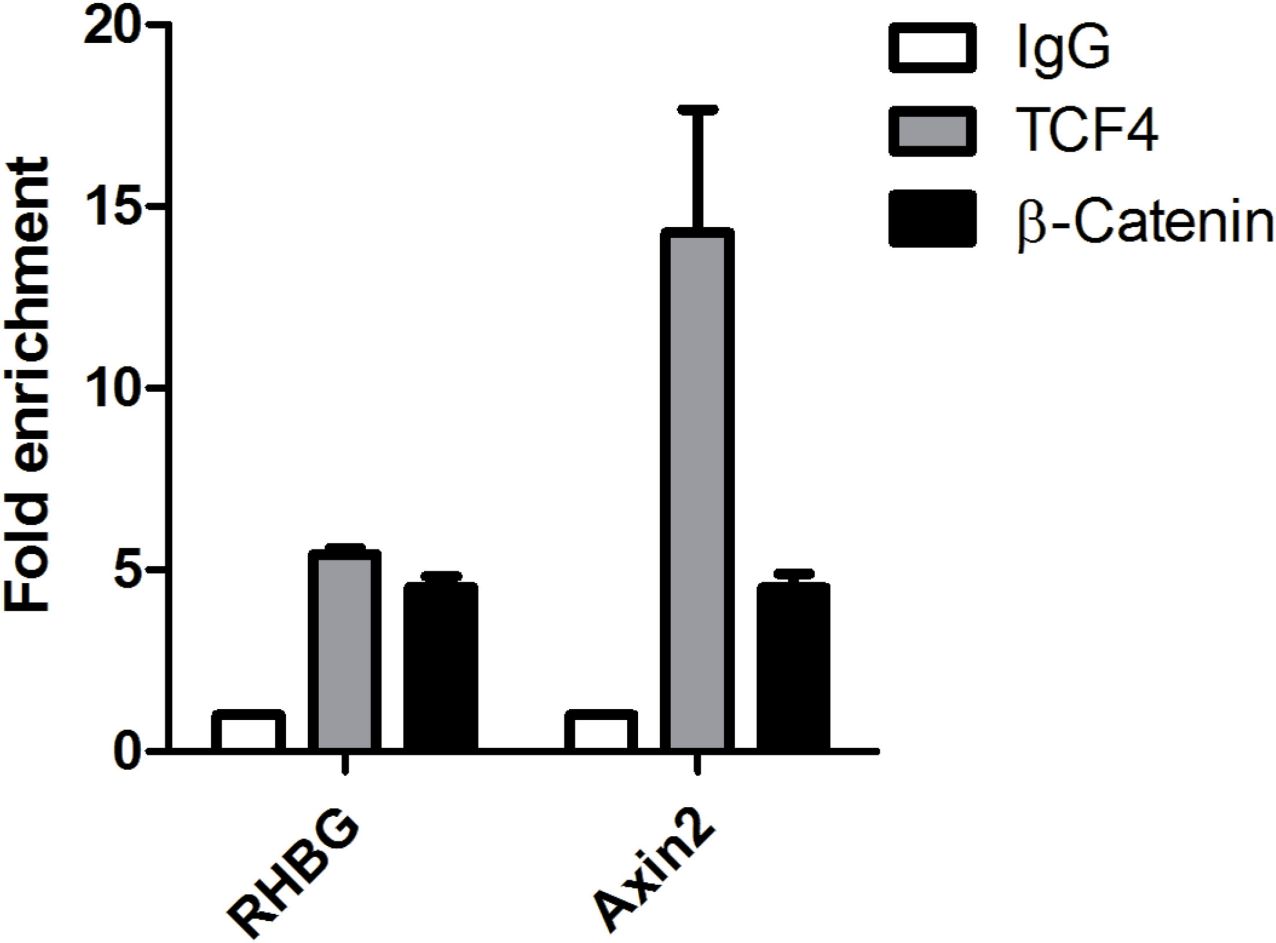
TCF4/β-catenin binds to the *RHBG* promoter. HepG2 cells were cross-linked with formaldehyde followed by chromatin digestion. Chromatin immunoprecipitations were performed using antibodies targeting either β-catenin, TCF4 or IgG, as a control. Purified DNA was analyzed by qPCR using the indicated primers. The amount of immunoprecipitated DNA with each antibody is represented as signal relative to IgG (equivalent to 1) (n = 2).

We finally performed chromatin immunoprecipitation (ChIP) assays to determine whether TCF4 and β-catenin are capable of binding the -60/+142 segment of *RHBG* promoter *in vivo*. Nuclear extracts obtained from the HepG2 cells were subjected to protein/DNA complex crosslinking and immunoprecipitation was performed using antibodies targeting either β-catenin, TCF4 or IgG, as a control. qPCR using primers within the H region reveal that TCF4 and β-catenin bind to this fragment of the *RHBG* promoter (Fig 9). Consistently, TCF4 and β-catenin did also bind to the *Axin2* promoter, taken as control, as previously reported [37,53].

These results indicate that TCF4/β-catenin specifically binds to the -60/+142 region of the *RHBG* promoter, and likely enhances *RHBG* expression in HepG2 cells.

## Discussion

This study identifies the hepatoma HepG2 cells as expressing the *RHBG* gene encoding an ammonium transport protein. We show that in these cells *RHBG* expression is largely dependent on β-catenin function. We perform a functional analysis of the *RHBG* upstream regulatory sequence, revealing a minimal region bearing a promoter activity. Our data indicate that the *RHBG* regulatory sequence is a TATA-less GC-rich promoter. We show that β-catenin and TCF4 are both able to bind the minimal promoter region *in vivo* and characterize potential TCF4 binding motifs important for the promoter activity. Our data support a direct role of β-catenin/TCF4 in the regulation of *RHBG* expression in this cell line, and further indicate that *RHBG* could serve as a direct reporter of the Wnt/β-catenin pathway in specific cancer cell contexts.

Hepatocellular carcinoma is the most common adult liver malignancy and many lines of evidence associate hyperactivation of the Wnt/β-catenin pathway to its initiation and development [56]. Abnormal activation of Wnt/β-catenin signaling, due to loss-of-function mutations in APC or activating mutations in β-catenin has been linked to various human malignancies including melanoma, breast, and colon carcinomas [22]. For instance, more than 80% of colon cancers bear truncations in APC, resulting in active β-catenin accumulation in the nucleus, the initial stage of transformation [56–58]. We show that *RHBG* is expressed in the colon cancer SW480 cells bearing an APC mutation and that its expression is also dependent on TCF4/ β-catenin. In contrast, the expression of the gene encoding the second non erythroid ammonium transport protein, RhCG, is independent of β-catenin signaling in either HepG2 or SW480 cells.

Our data are consistent with the *RHBG* overexpression observed in hepatocarcinoma obtained from surgical resections and bearing activating mutations in the β-catenin gene *CTNNB-1* [18]. *RHBG* was up-regulated in 9 over 10 HCC of the latter resections compared to normal liver, while it was slightly overexpressed in only one over 15 HCC showing wild-type *CTNNB-1*. *RHBG* overexpression was strongly correlated with upregulation of *GLUL*, but also of *SLC13-A3* encoding a sodium-dicarboxylate (including α-ketoglutarate and succinate) transporter and *GPR49*, also known as LGR5, a coreceptor of Wnt signaling. A correlation between HCC with activated β-catenin pathway and upregulation of GS, of ornithin amino transferase (involved in glutamate synthesis), and of the Glt1 glutamate transporter, were also reported [31]. The upregulation of GS suggests that HepG2 cells, and possibly specific HCC, could be able to adapt their metabolism to favor glutamine synthesis from glutamate and ammonium, a function restricted to perivenous hepatocytes in normal liver. For instance, in mouse, the Wnt/β-catenin pathway has been shown to play a key role in liver zonation [19,20]. This process ensures a functional specialization of hepatocytes along the porto-central axis of the liver lobule and determines the fate of periportal hepatocytes, active in urea synthesis, or perivenous hepatocytes, active in glutamine synthesis for instance. Mouse Rhbg is specifically present at the cell surface of the latter hepatocytes and co-localizes with GS [59]. HepG2 cells were shown to have a reduced activity of the urea cycle [60]. However, it should be kept in mind that these cells show important plasticity of the metabolic networks according to the availability of key metabolite in the surrounding medium as glucose and insulin [61]. Of note, it was recently shown that HepG2 cells have a glutamine-addiction phenotype [34]. Addiction to glutamine is a metabolic particularity of many cancer cells showing concomitant high rates of glutamine transport and metabolism [62]. Proliferation of HepG2 was importantly reduced upon withdrawal of exogenous glutamine, and simultaneous drug-mediated inhibition of GS activity further hampered proliferation [34]. In conditions where glutamine synthesis would be favourable, it is tempting to hypothesize that correlated upregulation of RhBG could help to scavenge ammonium, providing one of the substrates of GS. Whether RhBG actively participates to cancer cell metabolism will require further investigation. Rh factors were shown to act as bidirectional ammonium transport proteins [8,63]. Rhcg is expressed at the apical membrane of specific epithelial kidney cells, together with the H^+^ V-ATPase which is supposed to drive NH_3_ efflux by favouring urinary trapping of NH_4_^+^ [12,13,17]. The GS activity could serve as a trapping system, driving ammonium influx via co-expressed Rh factors such as RhBG by consuming ammonium for glutamine generation. Interestingly, a corresponding mechanism exists in *E*. *coli* where the GS activity is strictly required to drive substrate uptake via the Rh orthologue AmtB [64]. However, to date, a role of Rhbg in the process of ammonium detoxification via glutamine synthesis has not been highlighted *in vivo*, as plasma levels of glutamine and urea appear normal in mice lacking Rhbg [15]. GS is the sole enzyme catalyzing glutamine synthesis. In addition to its presence in perivenous hepatocytes, a detailed analysis in mouse revealed that it is also highly expressed and active in the epididymis epithelial cells and in Leydig cells, the testosterone-producing cells in the testis [65]. Although the physiological role of GS in these cells is unknown, it should be noted that Rh factors are also co-expressed [17,66].

Ammonium was recently proposed to play a particular role in a tumoral context [67]. Up-regulated glutaminolysis in glutamine-addicted cancer cells results in NH_3_ production. The latter molecule was shown to act as an autocrine and paracrine diffusible signal that triggers a specific autophagic program, in turn enabling survival and proliferation of cancer cells deep in a tumour mass [67,68]. Whether Rh factors could play a role in these processes by participating to trans-cellular ammonium movements remains to be evaluated.
